# Rapid screening of innate immune gene expression in zebrafish using reverse transcription - multiplex ligation-dependent probe amplification

**DOI:** 10.1186/1756-0500-4-196

**Published:** 2011-06-15

**Authors:** Janneke Rotman, Walter van Gils, Derek Butler, Herman P Spaink, Annemarie H Meijer

**Affiliations:** 1 BaseClear B.V., Einsteinweg 5, 2333CC, Leiden, The Netherlands; 2 Institute of Biology, Leiden University, Einsteinweg 55, 2333 CC, Leiden, The Netherlands

## Abstract

**Background:**

With the zebrafish increasingly being used in immunology and infectious disease research, there is a need for efficient molecular tools to evaluate immune gene expression in this model species. RT-MLPA (reverse transcription - multiplex ligation-dependent probe amplification) provides a sensitive and reproducible method, in which fluorescently labelled amplification products of unique lengths are produced for a defined set of target transcripts. The method employs oligonucleotide probes that anneal to adjacent sites on a target sequence and are then joined by a heat-stable ligase. Subsequently, multiplex PCR with universal primers gives rise to amplicons that can be analyzed with standard sequencing equipment and relative quantification software. Allowing the simultaneous quantification of around 40 selected markers in a one-tube assay, RT-MLPA is highly useful for high-throughput screening applications.

**Findings:**

We employed a dual-colour RT-MLPA probe design for chemical synthesis of probe pairs for 34 genes involved in Toll-like receptor signalling, transcriptional activation of the immune response, cytokine and chemokine production, and antimicrobial defence. In addition, six probe pairs were included for reference genes unaffected by infections in zebrafish. First, we established assay conditions for adult zebrafish infected with different strains of *Mycobacterium marinum *causing acute and chronic disease. Addition of competitor oligonucleotides was required to achieve peak heights in a similar range for genes with different expression levels. For subsequent analysis of embryonic samples it was necessary to adjust the amounts of competitor oligonucleotides, as the expression levels of several genes differed to a large extent between adult and embryonic tissues. Assay conditions established for one-day-old *Salmonella typhimurium*-infected embryos could be transferred without further adjustment to five-day-old *M. marinum*-infected larvae. RT-MLPA results were compared with results of previous transcriptome analyses and with real-time PCR data, demonstrating a good correlation between all expression analysis methods.

**Conclusions:**

The RT-MLPA assay developed in this study provides a rapid, cheap, and robust analysis tool for simultaneous quantification of a set of 34 innate immune response genes. With adjustment of conditions, the assay is suitable for infection studies in both adult and embryonic zebrafish. Application of RT-MLPA will facilitate high-throughput screening of immune responses in the zebrafish model.

## Background

The use of zebrafish models in immunological and infectious disease research has rapidly expanded over the recent years [1,2]. Important advantages of zebrafish include its amenability to large-scale forward and reverse genetic screening and small molecule library screening [3,4]. The recent development of a robotic system for bacterial infection of zebrafish embryos will facilitate antimicrobial drug screening in this model at high-throughput level [5]. The availability of robust molecular tools for the rapid evaluation of immune responses is a highly desired addition to zebrafish-based screening approaches.

Multiplex Ligation-dependent Probe Amplification (MLPA) is a high resolution method to establish the copy number of around 40 nucleic acid sequences in a one-tube reaction [6,7]. The technique was initially developed to determine copy number variation in genomic DNA, but has also been adapted for quantification of mRNA transcript levels [8]. Since its introduction in 2002, MLPA has rapidly become accepted in genetic diagnostic laboratories, where it is used for the detection of disease-associated polymorphisms, deletions, duplications and rearrangements in genomic DNA [7]. Reasons for the rapid adoption of MLPA in diagnostics are the relative simplicity, low costs (ca. 10 euro per reaction), high-throughput capacity, sensitivity, accuracy and robustness of the technique. The use of reverse-transcriptase-MLPA (RT-MLPA) for mRNA expression profiling is less common but has proven useful for example in human immunology and cancer research [9,10]. In contrast to approximately 250 MLPA kits available for DNA analysis, only three RT-MLPA kits (including a human inflammation probe set, a human apoptosis probe set, and a mouse inflammation probe set) are commercially available (MRC Holland, http://www.mlpa.com). While the application of existing RT-MLPA tests is fast and simple, the development of new tests for detection of other sets of mRNAs is a significant investment.

**Figure 1:**
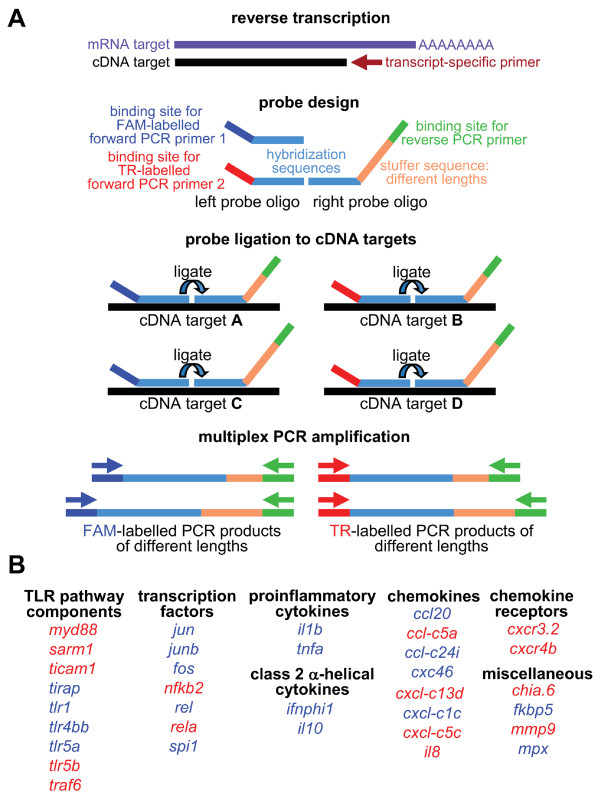
**Two-colour RT-MLPA assay**. (A) Schematic representation of the RT-MLPA assay design (figure adapted from MRC-Holland, http://www.mlpa.com). (B) Composition of the zebrafish immune response RT-MLPA assay. Expression of genes indicated in blue and red is detected by FAM-labelling and TR-labelling, respectively. In addition, three reference genes (*bactin1*, *ppial*, *rplp0*) are included both in the probe set for FAM-labelling and in the probe set for TR-labelling.

Setting up of an RT-MLPA test for a new set of mRNAs requires first the design of oligonucleotide probes. Detection of each different target sequence requires two oligonucleotide probes, designed such that they anneal to immediately adjacent sites on a target cDNA sequence produced by reverse transcription of mRNA (Figure 1A). One of the two oligonucleotide probes contains a stuffer sequence of variable length. Following hybridization to the target sequence, the probe pair is ligated by a heat-stable ligase and amplified using fluorescently labelled universal primers. The fact that only one primer pair is used, is the prerequisite for simultaneous reproducible amplification of different sequences and makes relative quantification possible [6,8]. Oligonucleotide probes that are not ligated will not be amplified. Due to use of different stuffer sequences, amplification of each ligated oligonucleotide probe pair gives rise to an amplification product of unique length. The amplicons are separated using the standard electrophoresis equipment that is also used for sequence analysis and peaks are quantified using relative quantification software.

Probes used in MLPA usually range between 80 to 400 nucleotides in length. Accurate chemical synthesis of MLPA probes is possible up to a length of approximately 180 nucleotides, while M13 cloning (using SALSA vectors, MRC Holland, http://www.mlpa.com) is used to produce probes up to 400 nucleotides in length [6,7,11]. To bypass the laborious M13 cloning procedures, a dual-colour approach has been applied [12]. In this approach, two universal primer pairs are used that give similar amplification efficiencies but are labelled with different fluorophores, thereby increasing the maximum possible number of synthetic probe pairs in a single MLPA assay.

Following probe design, a second time-consuming step in development of an RT-MLPA assay is optimization of the assay conditions by addition of competitor oligonucleotides (probes without universal primer sequences). Competitor oligonucleotides need to be used to titrate down the signals of genes with higher expression levels than others, such that all amplicons will produce peak heights in a similar range [8]. Titration conditions may need to be adjusted separately for different sample types, for example for different tissues or for samples from experimental manipulations that lead to large differences in gene expression. Therefore, RT-MLPA is most useful for high-throughput applications that involve repetitive analysis of a targeted transcript set in similar biological samples.

Here we describe the development of a first RT-MLPA assay for application in zebrafish models. The probe set targets 34 genes with functions in the innate immune system and contains 6 probe pairs for reference genes that are not affected under infection conditions. We show that a dual-colour approach previously developed for MLPA [12] can also be adopted for RT-MLPA, allowing us to combine 41 synthetic probe pairs in a single assay. We tested the method using samples from *Mycobacterium marinum *and *Salmonella typhimurium *infection experiments and established assay conditions for analysis of both adult and embryonic tissues. RT-MLPA results correlated well with results from previous transcriptomics studies and with results from real-time PCR analysis.

## Methods

### MLPA probe and RT-primer design

MLPA probes and RT-primers were designed for target mRNAs of 34 immune response genes and three reference genes (Additional file 1). For each target a pair of probes was designed, binding adjacently to each other on the target sequence, with the resulting ligation site located maximally 7 nucleotides from an exon-exon boundary. In addition, a control probe pair, designed on an intron sequence of one of the targets (*cxcr3.2*), was included in the set to check for the interference of genomic DNA in the MLPA run. The probe oligos were designed following the guidelines described in the manual "Designing synthetic MLPA probes" (http://www.mlpa.com, MRC Holland, Amsterdam, The Netherlands). All probe pairs contain the same universal binding site for the reverse PCR primer on the right probe oligo (RPO). The left probe oligo (LPO) for approximately half of the immune response genes harbours a sequence corresponding with forward PCR primer 1 labelled with FAM, while the remaining LPOs harbour a different sequence corresponding with forward PCR primer 2 labelled with Texas Red (TR). Two different probe pairs were designed for each of the three reference genes, one for amplification with FAM-labelled primer 1 and one for amplification with TR-labelled primer 2, thus resulting in a total of six RT-MLPA reference sequences. The probe pairs were designed such that the fragments resulting from PCR amplification are spaced by a minimum of three nucleotides in a range from 91-178 base pairs. Additionally, competitor oligonucleotides were created, consisting of the left probe oligo hybridization sequence (LHS), but lacking the universal primer sequence. A synthetic control DNA template was generated for each target and used to test the probe pairs for efficiency in setting up the RT-MLPA assay and as a positive control during runs. RT-Primers, reverse complement to the strand corresponding to the probe sequence, were designed for all target mRNAs, and were positioned immediately adjacent to the actual MLPA probe with no more than 15 nucleotides between the last nucleotide of the primer and the first nucleotide of the probe sequence, and with a maximum overlap of 7 nucleotides. The RT primers all have a Tm in the range of 55-65 °C and a GC content between 35 and 60%.

### RT-MLPA assay

RT-MLPA reactions were performed using SALSA^® ^MLPA^® ^Reagents kit EK1-RT (MRC-Holland, Amsterdam, The Netherlands) according to the manufacturer's instructions for RT-MLPA reactions (version 10, 18-09-2008). All reaction steps were performed in a thermocycler with heated lid (105°C) using 0.2 ml thin-walled PCR tubes. RPOs were treated with T4 polynucleotide kinase (New England Biolabs) before use to establish 5' phosphorylation. A mix of RT-primers and dNTPs was prepared containing 1 pmol/μl of the RT primer for each target transcript and 2.5 mM of dNTPs. The RT primer/dNTP mix (1 μl) was added to the RNA sample (100 ng total RNA in 2.5 μl) and SALSA RT buffer (1 μl) at 4°C. This mixture was subsequently heated for 1 min to 80°C and incubated for 5 min at 45°C. Subsequently, the temperature was cooled down to 37°C, 30 units of M-MLV Reverse Transcriptase in SALSA dilution buffer (1.5 μl) were added, and the RT reaction was performed for 15 min at 37°C. The RT enzyme was then inactivated by heating to 98°C for 2 min and the mixture was cooled down to 25°C. A probe mix containing all probe oligos was prepared containing 2 fmol/μl of each oligo and, if necessary, competitor oligos were added to the required ratio (Additional file 2). The probe mix (1.5 μl) and MLPA buffer (1.5 μl) were added to the RT reaction, and after heating for 1 min at 95°C, the mixture was incubated for 16 h at 60°C to let the LPO and RPO probes hybridize to their respective targets. Subsequently, the mixture was cooled down to 54°C, 32 μl of Ligase-65 mix was added, and ligation of the LPO and RPO probes was performed by incubating for 15 min at 54°C. The ligase enzyme was inactivated by heating to 98°C for 2 min and the mixture was cooled down to 4°C. Next, 10 μl of the ligation reaction was mixed with 4 μl of SALSA PCR buffer and 26 μl water. When the tubes were in the thermal cycler at 60°C, 10 μl of polymerase mix was added containing 2 μl of PCR primer mix (10 pmol/μl of each forward primer and 20 pmol/μl of the reverse primer), 2 μl SALSA Enzyme Dilution Buffer, 5.5 μl water, and 0.5 μl SALSA Polymerase. The PCR reaction was carried out with slight modifications to the manufacturer's protocol to provide higher selectivity of the PCR primers. To this end the amplification temperature was increased to 65°C, and the number of cycles was increased to 38, resulting in the following cycling conditions: 30 s at 95°C; 30 s at 65°C; 1 min at 72°C. PCR cycling was ended with 20 min incubation at 72°C, after which the reaction was cooled down to 4°C. After PCR, the resulting DNA fragments were treated for 10 min at 37°C with 5 μl of Polymerase I Large Klenow fragment (Promega Corp., Madison, WI, USA) diluted in a 2.5 mM dNTP mix to correct for strand overhang. Next, the fragments were column-purified with the ZR DNA Sequencing Clean-up Kit™ (Zymo Research Corporation, Orange, CA, USA), according to the manufacturer's instructions, to prevent for dye-blobs in capillary fragment analysis. After PCR purification, 0.7 μl of sample was mixed with 0.3 μl of GS500-LIZ size standard and fragment analysis was performed on a ABI 3100 sequencer in GeneScan mode. Data were analyzed using GeneMarker^® ^software, version 1.7 (SoftGenetics, LLC. State College, PA, USA). Raw data were imported with the Analysis Type set on 'Fragment (Animal) Analysis' and the following default settings for the Data Process Options: Raw Data Analysis: auto range, smooth, peak saturation, baseline subtraction, pull-up correction, spike removal; Size call: Local Southern; Allele Call: auto range 60-600 with peak detection threshold settings at: intensity > 100, percentage >5, local region > 25%, max call intensity 30000; Stutter peak filter %: left 90, right 40; Plus-A filter. Data were scaled using the GS500-LIZ size standard and peaks of target transcripts were assigned using a template with the fragment sizes of the RT-MLPA amplification products. A table containing the heights of each assigned peak scored in arbitrary units was exported and further analysed in Microsoft Excel. For normalization of the data, the peak heights of the target transcripts were divided by the average peak heights of the reference genes included in the same dye group. Based on parallel runs of the synthetic DNA control template described above there was no need to correct for possible signal sloping. Fold change values were calculated by dividing the normalized peak values of a treatment sample by the normalized peak values of the control sample.

### Real-time PCR

From 1 μg of total RNA cDNA was synthesized using the I-Script cDNA synthesis Kit (Bio-Rad Laboratories, Hercules, CA, USA). For real-time PCR assays SYBR Green Universal PCR Mastermix 2x (Applied Biosystems, Foster City, CA, USA) was used. PCR primers were designed with Primer Express Software version 1.7 (Applied Biosystems, Foster City, CA, USA). For 33 of the 40 RT-MLPA target transcripts, including all six reference sequences, real-time PCR primer sets were developed, covering the hybridization sequence of each RT-MLPA target (Additional file 1). Each real-time PCR reaction contained 12,5 μl 2X PCR Mastermix, forward and reverse primer in a final concentration of 300nM, 1 μl of cDNA template and the volume adjusted to 25 μl with ddH_2_O. The reactions were run on an ABI PRISM™ 7500 Sequence Detection System (Applied Biosystems). The solution was subjected to a protocol of subsequently 95 °C for 10 minutes, followed by 40 cycles of 15 s at 95 °C and 1 min. at 60 °C. All reactions were performed in duplicate. Relative quantification was performed according to Vandesompele et al. [13] using three reference genes (coding for tyrosine 3-monooxygenase activation protein (NM_201484), NADH dehydrogenase (AC024175), and 16S ribosomal RNA (CK739347) that were selected from the GeNorm Zebrafish reference kit (PrimerDesign Ltd, Southampton, UK) for normalization.

### RNA samples

Total RNA samples from zebrafish adults infected with *M. marinum *E11 and Mma20 strains and PBS-injected control fish were identical to those used in a previous microarray study [14]. Three biological replicates of infected fish and controls were used for RT-MLPA analysis, similar as in the previous microarray study. Zebrafish embryos at 28 hours post fertilization (hpf) were injected into the caudal vein with approximately 200 colony forming units (CFU) of *S. typhimurium *infection or injected with PBS as a control. Total RNA was isolated from pools of 15-20 embryos per treatment group at 8 hours post infection (hpi) and subjected to microarray analysis as previously described [15]. Samples were analyzed from three independent *S. typhimurium *infection experiments. Zebrafish embryos at 48 hpf were injected into the yolk sac with approximately 2000 CFU of *M. marinum *E11 bacteria in PVP carrier solution or with carrier solution alone as a control [5]. Total RNA was isolated from pools of 15-20 embryos at 3 days post infection (dpi).

## Results

### Development of an RT-MLPA probe set for the zebrafish innate immune response

For rapid screening of innate immune responses in zebrafish we set out to develop an RT-MLPA assay. We adopted a two-colour assay design, which was previously used in MLPA applications for DNA diagnostics [12]. This two-colour design, using FAM (blue) and TR (red) dyes for multiplex PCR amplification of MLPA probes, allowed combining 41 chemically synthesized MLPA probes (size range: 91 to 178 oligonucleotides) in a single reaction (Figure 1A, Additional file 1). Three reference genes, *bactin1*, *ppial*, and *rplp0*, were selected based on previous transcriptomics data showing unchanged expression levels of these genes during *M. marinum *infection of adult zebrafish and *S. typhimurium *infection of embryos [14-19]. Two different probe pairs were designed for each of the three reference genes, one for amplification with FAM-label and one for amplification with TR-label. As an internal control for possible genomic DNA contamination, a probe pair designed on an intron sequence of one of the target genes was included in the probe set for amplification with FAM-label. A total of 34 genes involved in innate immunity were selected for probe design (Table 1). The selection included: 9 genes involved in TLR signalling; 7 immune-related transcription factor genes; 4 genes for pro-inflammatory cytokines and class II α-helical cytokines; 8 genes for chemokines that we have found to be induced by infections in previous transcriptomics studies [14-19]; 2 genes for chemokine receptors; and 4 miscellaneous genes involved in inflammation and microbial defense, including matrix metalloproteinase 9 (*mmp9*), myeloperoxidase (*mpx*), a homolog of human acidic chitinase (*chia.6*), and the gene for FK506 binding protein 5 (*fkbp5*), which is a marker for activation of glucocorticoid receptor signalling. These selected genes were divided over the probe sets for amplification with FAM-label and TR-label (Figure 1B, Additional file 1).

**Table 1:** Target genes of the zebrafish immune response RT-MLPA assay

**Gene name**^**1**^	Description	ZFIN ID	GenBank Accession	Entrez Gene ID	**UniGene ID**^**2**^
***Immune response genes***					
*ccl20a*	chemokine ccl-c24a (si:dkey-150o13.1, ccl20)	ZDB-GENE-091204-95	NM_001113595	563152	Dr.133624
*ccl-c24i*	transcribed locus, weakly similar to NP_001108573.1 chemokine CCL-Cub	-	CN021049	-	Dr.125570
*ccl-c5a*	chemokine ccl-c5a (si:ch211-89f7.4)	ZDB-GENE-060526-181	NM_001082906	794891	Dr.133987
*chia.6*	chitinase, acidic.6	ZDB-GENE-030131-1140	NM_199603	322420	Dr.77223
*cxc46*	cxc chemokine 46	ZDB-GENE-090303-1	XM_002664187	100321314	Dr.117585
*cxcl-c13d*	chemokine cxcl-c13d	-	NM_001113651	100003911	Dr.92011
*cxcl-c1c*	chemokine cxcl-c1c (si:ch73-6k14.1)	ZDB-GENE-090313-165	NM_001115060	795785	Dr.113696
*cxcl-c5c*	chemokine cxcl-c5c	-	NM_001115055	567537	Dr.111760
*cxcr3.2*	chemokine (C-X-C motif), receptor 3.2	ZDB-GENE-041114-186	NM_001007314	791973	Dr.82754
*cxcr4b*	chemokine (C-X-C motif), receptor 4b	ZDB-GENE-010614-1	NM_131834	114447	Dr.75485
*fkbp5*	FK506 binding protein 5	ZDB-GENE-030616-630	NM_213149	368924	Dr.78793
*fos*	v-fos FBJ murine osteosarcoma viral oncogene homolog	ZDB-GENE-031222-4	NM_205569	394198	Dr.12986
*ifnphi1*	interferon phi 1	ZDB-GENE-030721-3	NM_207640	360134	Dr.85981
*il10*	interleukin 10	ZDB-GENE-051111-1	NM_001020785	553957	Dr.135567
*il1b*	interleukin 1, beta	ZDB-GENE-040702-2	NM_212844	405770	Dr.30443
*il8*	interleukin 8	ZDB-GENE-081104-317	CT826376	100002946	Dr.112992
*jun*	v-jun sarcoma virus 17 oncogene homolog (c-Jun)	ZDB-GENE-030131-7859	NM_199987	335916	Dr.1064
*junb*	jun B proto-oncogene	ZDB-GENE-040426-2172	NM_213556	407086	Dr.10326
*mmp9*	matrix metalloproteinase 9	ZDB-GENE-040426-2132	NM_213123	406397	Dr.76275
*mpx*	myeloid-specific peroxidase	ZDB-GENE-030131-9460	NM_212779	337514	Dr.75725
*myd88*	myeloid differentiation primary response gene (88)	ZDB-GENE-040219-3	NM_212814	403145	Dr.134592
*nfkb2*	nuclear factor of kappa light polypeptide gene enhancer in B-cells 2, p49/p100	ZDB-GENE-030131-6701	NM_001001840	415100	Dr.117553
*rel*	v-rel reticuloendotheliosis viral oncogene homolog	ZDB-GENE-040718-255	NM_001001841	415101	Dr.86023
*rela*	v-rel reticuloendotheliosis viral oncogene homolog A	ZDB-GENE-040825-4	NM_001001839	415099	Dr.84126
*sarm1*	sterile alpha and TIR motif containing 1	ZDB-GENE-040219-1	NM_001130596	403143	Dr.84799
*spi1*	spleen focus forming virus (SFFV) proviral integration oncogene spi1 (pu.1)	ZDB-GENE-980526-164	NM_198062	30117	Dr.34508
*ticam1*	toll-like receptor adaptor molecule 1 (ticam1, trif)	ZDB-GENE-040219-2	NM_001044759	403147	Dr.82215
*tirap*	toll-interleukin 1 receptor (TIR) domain containing adaptor protein (tirap, mal)	ZDB-GENE-040219-4	AL916644	403148	Dr.87438
*tlr1*	toll-like receptor 1	ZDB-GENE-040220-1	NM_001130593	403127	Dr.89709
*tlr4bb*	toll-like receptor 4b, duplicate b	ZDB-GENE-040219-9	NM_212813	403132	Dr.89442
*tlr5a*	toll-like receptor 5a	ZDB-GENE-040219-14	AY389449	403138	Dr.89423
*tlr5b*	toll-like receptor 5b	ZDB-GENE-040219-15	NM_001130595	403139	Dr.89707
*tnfa*	tumor necrosis factor a (TNF superfamily, member 2)	ZDB-GENE-050317-1	NM_212859	405785	Dr.89727
*traf6*	TNF receptor-associated factor 6	ZDB-GENE-030131-5735	NM_001044752	554561	Dr.74618
					
***Reference genes***					
*bactin1*	beta-actin 1	ZDB-GENE-000329-1	NM_131031	57934	Dr.35143
*ppial*	peptidylprolyl isomerase A, like (cyclophilin)	ZDB-GENE-030131-7459	NM_199957	335519	Dr.78109
*rplp0*	acidic ribosomal protein (ARP)	ZDB-GENE-000629-1	NM_131580	58101	Dr.55617

^1 ^Chemokine genes *ccl-c5a*, *ccl-c24i*, *cxcl-c5c*, *cxcl-c1c*, and *cxcl-c13d *are named by the chromosome on which they are located according to Nomiyama et al. [21]. All other gene names are according to ZFIN (http://www.zfin.org).

^2 ^UniGene IDs are according to Build #122.

### RT-MLPA analysis of Mycobacterium-infected zebrafish adults

**Figure 2:**
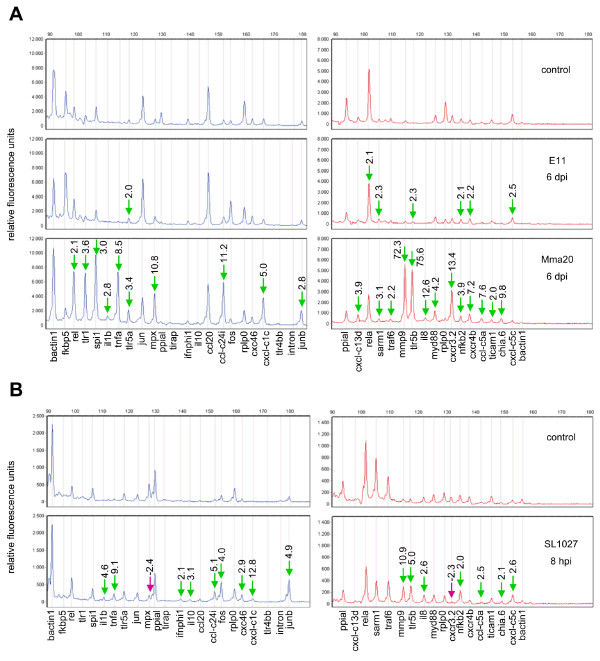
**RT-MLPA assays of *M. marinum*-infected zebrafish adults and *S. typhimurium*-infected zebrafish embryos**. (A). Zebrafish were infected with *M. marinum *strains E11 and Mma20, or injected with PBS as a control, and samples were taken at 6 dpi [14]. (B) One-day-old zebrafish embryos (27 hpf) were intravenously infected with *S. typhimurium *strain SL1027, or injected with PBS as a control, and samples were taken at 8 hpi (35 hpf). Three biological replicates were analyzed in both experiments. A representative example of the RT-MLPA assay result of each is shown. Peak patterns of the FAM-labelled amplification products are in blue and peak patterns of the TR-labelled amplification products are in red. Fold change values of amplification products that were more than 2-fold up-regulated in infected zebrafish compared to the uninfected control are indicated with green arrows, and fold change values of amplification products that were more than 2-fold down-regulated are indicated with purple arrows. Fold change values are based on the combined data from three biological replicates.

First we tested the RT-MLPA assay on samples from a previous *M. marinum *infection study of adult zebrafish [14]. In this study, two different strains of *M. marinum*, E11 and Mma20, were used that cause chronic and acute infection, respectively, and lead to different gene expression profiles. At 6 dpi, the acute infection caused by Mma20 infection was accompanied by induced microarray expression levels of the majority of genes included in the RT-MLPA assay. Therefore, RNA samples from this infection condition were chosen to set up the RT-MLPA assay conditions. Relative quantification in RT-MLPA analysis requires the use of competitor oligonucleotides to titrate down the signals of genes with higher expression levels than others. Following titration, RT-MLPA analysis of the Mma20 infection sample at 6 dpi showed detectable peak heights for all the reference genes and immune response genes in the range between 100 and 11000 relative fluorescence units (RFU) (Figure 2). The intron control showed no detectable peak, indicating that there was no interference from genomic DNA contamination. The same assay conditions were then applied to test three biological replicates of E11 and Mma20 infections at 6 dpi, and the expression levels were analyzed relative to samples from PBS-injected control fish. The results showed higher induction levels in Mma20 infection than in E11 infection (Figure 2A), in agreement with the severity of the infections caused by the two different strains.

### RT-MLPA analysis of Salmonella- and Mycobacterium-infected zebrafish embryos

Next we performed RT-MLPA analyses on samples from infection studies in embryos. Initial tests showed that the assay conditions developed for samples from *M. marinum*-infected adult fish were not applicable to samples from embryos, because several genes showed strongly different basal or induced expression levels between adult and embryonic tissues. Therefore, titration levels of competitor oligonucleotides had to be adjusted for analysis of embryo samples. The titration was performed on samples from *S. typhimurium*-infected embryos at 8 hpi, a stage at which many of the genes included in the RT-MLPA probe set were known to be differentially expressed [15,16,19]. After establishment of appropriate assay conditions, *S. typhimurium*-induction of a broad set of immune response genes was detected, as well as repression of the *cxcr3.2 *and *mpx *genes (Figure 2B). A clear induction of several immune response genes was also observed in subsequent RT-MLPA analysis of a sample from five-day-old larvae that had been infected three days earlier by injection of *M. marinum *E11 bacteria into the yolk sac (Additional file 4). Therefore, the titration conditions set-up for one-day-old *S. typhimurium*-infected embryos were also suitable for analysis of *M. marinum *infection at a later developmental stage. However, it should be noted that the analysis of other developmental stages or other infection regimes, would still require a further assessment of the normalising function of the reference genes under those conditions.

### Comparison of RT-MLPA and transcriptomics data

The same RNA samples as those used for RT-MLPA analysis (Figure 2) were also subjected to microarray analysis and the data were used for comparison with the RT-MLPA results (Additional file 3).

Infection of adult fish with the *M. marinum *E11 strain was previously shown to lead to chronic infection with no visible disease symptoms at 6 dpi [14]. Of all 34 immune genes included the RT-MLPA set, only one (*cxcr4b*) showed a significant induction (2.1-fold) in the previous microarray analysis [14]. The induction of this gene was also above the 2-fold change threshold in the RT-MLPA analysis (2.2-fold), which additionally detected minor inductions (2.0-2.5-fold) of 6 other genes.

Infection with the *M. marinum *Mma20 strain was previously shown to cause acute disease symptoms at 6 dpi accompanied with high induction levels of many immune response genes [14]. In total 22 genes from the RT-MLPA gene set showed a significant change of 2-fold or higher in the microarray analysis. Similarly, 23 genes were above the 2-fold change threshold in the RT-MLPA analysis, of which 18 overlapped with those detected by microarray. All inductions above 3-fold in the microarray analysis (17 genes) were also detected by RT-MPLA, and, vice versa, the majority of inductions above 3-fold in RT-MLPA were also significant in the microarray analysis (15 out of 18 genes). Only the inductions of *sarm1 *(3.1-fold) and *myd88 *(4.2-fold) in RT-MLPA were not significant in the microarray results, and the high induction of *il8 *in RT-MLPA (12.6-fold) could not be verified due to its absence on the microarray platform. Eight genes were induced above 5-fold in both microarray or RT-MLPA, with *mmp9 *showing the highest induction in both techniques (58-fold in microarray; 72-fold in RT-MLPA). Other genes showing above 5-fold induction with only one of the two methods (7 genes excluding *il8*, which is absent on the microarray) also showed clear induction in the other method (2.8-3.9-fold). The largest quantitative difference between the results of the two methods was observed for *il1b*, which was induced 26.6-fold in the microarray analysis and 2.8-fold in RT-MLPA.

Microarray and RT-MLPA data of *S. typhimurium*-infected embryos were also in good agreement, not only showing similar gene induction profiles but also consistent down-regulation (approx. 2-fold) of *cxcr3.2 *and *mpx *expression (Figure 2B, Additional file 3). In total 16 genes in the microarray analysis and 18 in RT-MLPA showed fold-change differences above 2-fold, of which 13 were overlapping between the two methods. With the exception of *ccl20 *(4-fold induction in microarray), all other genes (9) showing above 3-fold change in the microarray analysis were also picked up by RT-MLPA. Vice versa, with the exception of *tlr5b *(5-fold induction in RT-MLPA), all other genes (8) with above 3-fold change in RT-MLPA were also significant in the microarray analysis. Although the induction of *tlr5b *was below the 2-fold change threshold, it showed 1.7-fold induction in the microarray analysis supported by 9 probes with a significant *P*-value (<0.0001).

In conclusion, both for *M. marinum *infection of adult fish and for *S. typhimurium *infection of embryos the results of microarray and RT-MLPA were largely consistent in that differential expression of the majority of genes tested was confirmed by both methods. As both methods are to be considered semi-quantitative and rely on entirely different technology and data processing, it is not surprising that differences in the absolute induction levels were observed. A possible explanation for some discrepancies is that the microarray and RT-MLPA are measuring different transcripts due to alternative splicing.

### Validation of RT-MLPA data by real-time PCR

**Figure 3:**
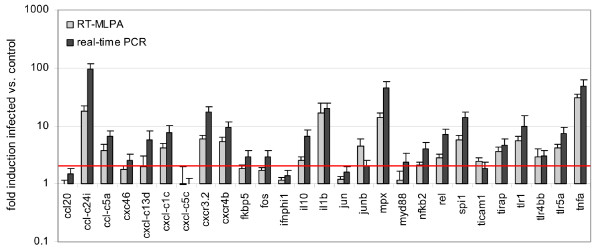
**Comparison of RT-MLPA and real-time PCR data**. An RNA sample from an adult zebrafish infected with *M. marinum *strain Mma20 at 6 dpi was compared to a sample from a PBS-injected control fish from the same experiment [14]. Real-time PCR assays were performed in duplicate and RT-MLPA runs were repeated four times. Data are plotted on a logarithmic scale. The red line is set at a 2-fold induction level. Error bars indicate the standard deviation.

To further confirm the RT-MLPA results we chose to perform real-time PCR quantification on RNA from one of the *M. marinum *Mma20-infected adult fish at 6 dpi. For real-time analysis we wanted to design PCR primers that overlapped with the hybridization sequence regions of the RT-MLPA probes. Good primer design in these regions was possible for 27 of the immune response genes and for the reference genes of the RT-MLPA assay. Three additional reference genes coding for tyrosine 3-monooxygenase activation protein, NADH dehydrogenase, and 16S ribosomal RNA (GeNorm zebrafish reference kit) were selected for normalization of the real-time PCR data. These reference genes and those from the RT-MLPA assay (*bactin1*, *ppial*, and *rplp0*) did not show significantly different expression between RNA samples from control and infected fish. Genes *ccl20*, *cxcl-c5c*, *ifnphi1*, and *jun *also did not shown significant induction in either real-time PCR or RT-MLPA. All other immune response genes tested showed 2-fold or higher induced expression levels in real-time PCR analysis of Mma20-infected fish compared to uninfected controls, except *ticam1 *which was just below the threshold (1.9-fold) (Figure 3). The majority of these genes also showed 2-fold or higher induction in RT-MLPA analysis. The most notable exception was *myd88*, which was not detectably induced in RT-MLPA compared to a 2.4-fold change in real-time PCR (Figure 3). In addition, three genes (*cxc46*, *fkbp5*, *fos*) with inductions between 2.6- and 3-fold in real-time PCR were just below the threshold in RT-MLPA (1.7-1.9-fold). While in general the absolute values of induction measured with real-time PCR were higher than those measured by the semi-quantitative RT-MLPA approach, the genes showing the highest induction levels in real-time PCR (*ccl-c24i*, *il1b*, *mpx*, and *tnfa*) also showed the highest inductions in RT-MLPA. In conclusion, real-time PCR confirmed the usefulness of the RT-MLPA assay for screening innate immune gene expression.

## Conclusions

In this work, we have developed an RT-MLPA analysis panel for relative quantification of innate immune gene expression in zebrafish. The method allows the rapid semi-quantitative expression screening of 34 genes involved in TLR signalling, transcriptional activation of the immune response, cytokine and chemokine production, and antimicrobial defence. Expression levels of these genes are determined relative to the expression of three reference genes in a single tube assay. The validity of the assay was demonstrated by comparison with transcriptomic data sets and real-time PCR analysis. Differential expression levels of the immune response genes detected with RT-MLPA covered a range between 2-fold and over 70-fold, similar to the results of microarray and real-time PCR analysis.

In development of the RT-MLPA assay we chose to employ a dual-colour labelling system previously used in DNA diagnostic MLPA applications [12]. In this system, two probe sets, each in the size range of approximately 90 to 180 oligonucleotides, are amplified using FAM-labelling and TR-labelling, respectively. In this size range it is possible to compose the RT-MLPA assay exclusively from synthetic probes, thus avoiding the laborious M13 cloning procedures used previously to extend the size range of probes for (RT-)MLPA analysis [6-8]. For future development it may be possible to further increase the number of testable mRNA targets by 50% with the use of a third label, which has also been reported in DNA diagnostic MLPA [20]. This would also facilitate further increasing the number of different reference genes in the assay.

The RT-MLPA assay was successfully applied to the analysis of RNA samples from infection experiments in adult and embryonic zebrafish. However, analysis of adult and embryonic material required different assay conditions. In RT-MLPA analysis it is necessary to compensate for large differences in expression levels of genes by the addition of competitor oligonucleotides to the probe mix [8]. We found that the basal and infection-induced expression levels of several genes differed to such an extent between adult and embryonic tissues that titration of the probe mix with competitor oligonucleotides had to be adjusted for these different developmental stages. Fortunately, assay conditions established for one-day-old *S.typhimurium*-infected embryos could be transferred without further adjustment to five-day-old *M. marinum*-infected larvae. Thus, by employing two types of titration conditions, we have been able to establish an RT-MLPA assay for infection studies in adult zebrafish and a second RT-MLPA assay that is applicable to infection studies in both embryos and larvae and with different pathogens.

Due to the fact that RT-MLPA is a relative quantification method, the composition of the probe mix cannot be easily altered without adjustment of the assay conditions. The replacement of one probe by another, or the addition of a probe for a new target transcript, will affect the peak heights of other transcripts. Therefore, especially if the new probe results in a large peak, this may require re-titrating the probe mix, as was also necessary when the sample-type was changed from adult to embryonic zebrafish. Since re-titration of the assay is a time-consuming step, RT-MLPA analysis is most suitable for recurrent analysis of the same set of target mRNAs under comparable experimental conditions. For such medium or high throughput screening applications, the innate immune response RT-MLPA assay developed in this study provides a rapid, cheap, and robust analysis tool.

## Competing interests

JR, WVG, and DB have contributed to this study as employees of BaseClear B.V., a company providing custom services in molecular biology, including MLPA analysis.

## Authors' contributions

JR and WVG designed the RT-MLPA probes, set-up the assays, performed RT-MLPA and real-time PCR experiments, and helped to draft the manuscript. DB supervised the RT-MLPA assay development and real-time PCR analysis. HPS and AHM conceived the study, selected the target transcripts, and performed the comparisons with transcriptome data. AHM wrote the manuscript and all authors read and approved the final version.

## Supplementary Material

Additional file 1 **Supplementary Table 1**. Oligonucleotide sequences used in RT-MLPA and real-time PCR. Click here for file

Additional file 2 **Supplementary Table 2**. Amounts of competitor oligonucleotides in the probe mixes for RT-MLPA analysis of zebrafish adults and embryos. Click here for file

Additional file 3 **Supplementary Table 3**. Comparison of RT-MLPA and microarray data. Click here for file

Additional file 4 **Supplementary Figure 1**. RT-MLPA assay of *M. marinum*-infected zebrafish embryos. Two-day-old zebrafish embryos (48 hpf) were injected in the yolk with *M. marinum *strain E11, or with PVP carrier solution as a control, and samples were taken at 3 dpi (5 dpf). RNA was isolated from pools of 15-20 embryos per treatment group. The RT-MLPA analysis was performed in triplicate and representative examples of the assay results are shown. Peak patterns of the FAM-labelled amplification products are in blue and peak patterns of the TR-labelled amplification products are in red. Fold change values of amplification products that were more than 2-fold up-regulated in infected zebrafish compared to the uninfected control are indicated with green arrows. Click here for file
