# Supplementary figures and images for: Rapid screening of innate immune gene expression in zebrafish using reverse transcription - multiplex ligation-dependent probe amplification
Source: BMC Res Notes. 2011 Jun 15;4:196. doi: 10.1186/1756-0500-4-196 (PMC3138464; doi:10.1186/1756-0500-4-196)

relative fluorescence units

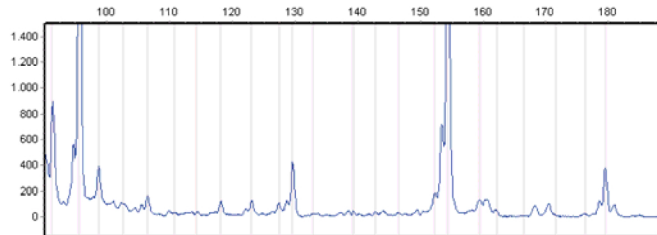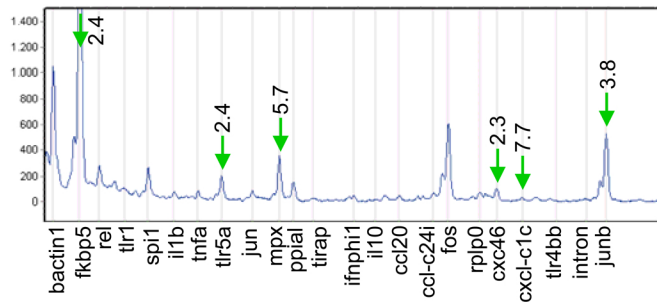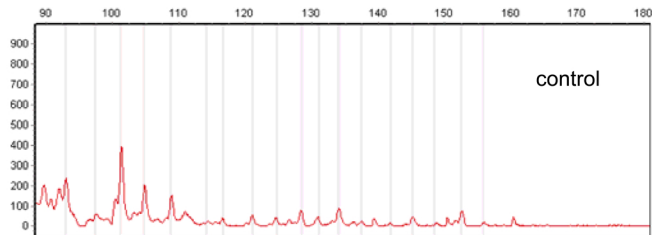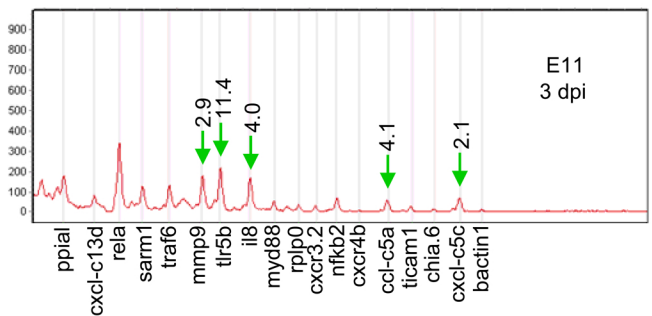

Supplement: Additional file 4 — Supplementary Figure 1. RT-MLPA assay of M. marinum-infected zebrafish embryos. Two-day-old zebrafish embryos (48 hpf) were injected in the yolk with M. marinum strain E11, or with PVP carrier solution as a control, and samples were taken at 3 dpi (5 dpf). RNA was isolated from pools of 15-20 embryos per treatment group. The RT-MLPA analysis was performed in triplicate and representative examples of the assay results are shown. Peak patterns of the FAM-labelled amplification products are in blue and peak patterns of the TR-labelled amplification products are in red. Fold change values of amplification products that were more than 2-fold up-regulated in infected zebrafish compared to the uninfected control are indicated with green arrows. [file 1756-0500-4-196-S4.PDF]
